# Isolation and identification of a potential new bovine viral diarrhea virus genotype associated with severe outbreaks in yaks (*Bos grunniens*) in China

**DOI:** 10.3389/fmicb.2026.1908819

**Published:** 2026-07-22

**Authors:** Xiji Ye, Chaohui Zhang, Yan Wang, Fuchao Zhang, Jinyu Xu, Xi Chen, Lin Xu, Jin Yu, Yongzhong Zeren, Changxiao Tian, Li Ren, Ni Song, Hua Yue, Cheng Tang

**Affiliations:** 1 Key Laboratory of Ministry of Education for Qinghai-Tibetan Plateau Animal Genetic Resource Reservation and Utilization, Southwest Minzu University, Chengdu, Sichuan, China; 2 Ganzi Animal Disease Prevention and Control Center, Kangding, Sichuan, China; 3 Sinovet (Jiangsu) Biopharmaceuticals Co., Ltd, Taizhou, Jiangsu, China

**Keywords:** bovine viral diarrhea virus, identification, new genotype, prevalence, yak

## Abstract

Bovine viral diarrhea virus (BVDV) is a globally significant pestivirus currently classified into three species: BVDV-1 (Pestivirus A), BVDV-2 (Pestivirus B), and BVDV-3 (HoBi-like, Pestivirus H). Here, we report the isolation and identification of a potential new BVDV genotype associated with severe outbreaks in yaks in China. Since 2024, an epidemic characterized by respiratory and gastrointestinal symptoms has been spreading across Sichuan and Qinghai Provinces. From January 2024 to October 2025, we collected 260 samples from 167 affected yaks from 14 farms. Among these, 55.69% of the yaks and all sampled farms tested positive for BVDV. Sequencing of the 5′ untranslated region (5′ UTR) and Npro fragments from clinical samples revealed that the dominant epidemic strains may represent a novel BVDV lineage. We isolated four cytopathic strains and successfully sequenced two genomes of strains SC-H03 and QH-B04, which share less than 77.8% nucleotide identity across the complete coding sequence (CDS) with well-defined BVDV-1/2/3 strains. Phylogenetic analyses of the complete CDS and individual genes placed SC-H03 and QH-B04 in a distinct monophyletic clade with strong bootstrap support. Pairwise genetic distances confirmed substantial divergence, and cross-neutralization assays revealed significant antigenic differences from BVDV-1 and BVDV-2 reference strains. In accordance with ICTV Pestivirus classification standards, these strains satisfy the criteria for a new Pestivirus species, which we propose to name BVDV-4 (Pestivirus T). Given its wide prevalence in yaks and association with high morbidity and mortality, our findings underscore the urgent need for targeted prevention and control measures.

## 1 Introduction

The genus Pestivirus, within the family Flaviviridae, encompasses viruses characterized by a broad host range, extensive genetic diversity, and potential for cross-species transmission (Simmonds et al., 2017; Smith et al., 2017). Advances in high-throughput sequencing have facilitated the discovery of novel pestiviruses in various livestock and wildlife species (Firth et al., 2014; Wu et al., 2018; Gao et al., 2020; Postel et al., 2021). According to the current taxonomy of the International Committee on Taxonomy of Viruses (ICTV), the genus Pestivirus currently includes 19 recognized species (Postel et al., 2021; Genus: Pestivirus | ICTV), several of which encompass economically important livestock pathogens such as Classical Swine Fever Virus (CSFV) and Bovine Viral Diarrhea Virus (BVDV), whose associated diseases are notifiable to the World Organisation for Animal Health (WOAH, formerly OIE).

BVDV is an important pathogen that imposes substantial production and economic burdens on the global cattle industry (Arnaiz et al., 2021). It exhibits a broad host range, including domestic cattle, domestic small ruminants, wild ruminants, and pigs (Rana et al., 2025a, 2025b). Clinical manifestations include acute enteric and respiratory syndromes, reproductive disorders, and the generation of persistently infected (PI) animals (Pinior et al., 2019; Al-Kubati et al., 2021). Based on cytopathic effects (CPE) in cell culture, BVDV isolates are classified into cytopathic (cp) and non-cytopathic (ncp) biotypes (Bolin and Grooms, 2004). Phylogenetic analysis classifies BVDV into three species: BVDV-1 (Pestivirus A), BVDV-2 (Pestivirus B), and BVDV-3 (HoBi-like viruses, Pestivirus H). Each species can be further subdivided into multiple subgenotypes according to sequence variation within conserved genomic regions, particularly the 5′ untranslated region (5′ UTR), Npro, and E2 genes, although no universally accepted criteria for subgenotype delimitation have yet been established (Yeşilbağ et al., 2017; Al-Kubati et al., 2021). The pronounced genetic and antigenic heterogeneity among these genotypes and subgenotypes poses substantial challenges to diagnosis, vaccine efficacy, and long-term disease control (Riitho et al., 2020).

The genome of BVDV comprises a single-stranded, positive-sense RNA molecule approximately 12.3 kb in length, featuring a 5′ UTR, a single open reading frame (ORF), and a 3′ UTR (Al-Kubati et al., 2021). The highly conserved 5′ UTR commonly serves as a target for detection and preliminary genotyping (Giangaspero and Harasawa, 2014). The ORF encodes a large polyprotein that undergoes co- and post-translational processing by cellular and viral proteases to yield four structural proteins (C, Erns, E1, and E2) and eight non-structural proteins (Npro, p7, NS2, NS3, NS4A, NS4B, NS5A, and NS5B) (Al-Kubati et al., 2021; Chi et al., 2022). Among these, the envelope glycoprotein E2 is the principal surface antigen and a critical determinant of virus–host interactions. E2 mediates viral attachment and entry and induces neutralizing antibody production, extensive amino-acid variability in surface-exposed regions and glycosylation motifs drives antigenic drift and serological diversity, thereby shaping genotype-specific immune recognition and limiting cross-protection afforded by existing vaccines (Bauermann et al., 2012; Gómez-Romero et al., 2023; Li et al., 2025b).

The yak (*Bos grunniens*) is a unique bovine species adapted to high-altitude environments and is crucial for livestock production among local herders. The global yak population is approximately 17 million head, distributed across more than 10 countries, including China, Mongolia, Bhutan, Nepal, India, and Russia, with the majority inhabiting the Qinghai-Tibet Plateau in China, which has an average altitude exceeding 3,500 meters (Ayalew et al., 2021). Several serological surveys indicated high prevalence of BVDV in yaks (Gao et al., 2013), and subgenotypes including 1a, 1b, 1d, 1q, 1m, and 1u have been reported in yaks to date (Gong et al., 2014; Deng et al., 2015; Li et al., 2025a). However, due to the harsh climate, inconvenient transportation, and fragmented veterinary services in the Qinghai-Tibet Plateau, systematic epidemiological data on BVDV remain limited, particularly regarding molecular prevalence and genetic diversity.

Since January 2024, an epidemic characterized by severe respiratory and gastrointestinal manifestations has been emerging and spreading in yaks across Sichuan and Qinghai provinces, with high morbidity and mortality in affected herds and producing substantial local economic losses. The purpose of this study was to investigate the pathogen characteristics of this epidemic. Unexpectedly, our results revealed that a novel BVDV has emerged and is prevalent in yaks, and its genome and antigenicity differ substantially from those of existing BVDV-1/2/3. Based on ICTV classification criteria for the genus Pestivirus, the novel BVDV identified in this study may represent a potential new BVDV genotype (Pestivirus T). Our findings expand our understanding of BVDV genetic diversity and have important implications for the surveillance, prevention, and control of BVDV in yaks.

## 2 Materials and methods

### 2.1 Specimen collection

Field investigations indicated that yaks of different ages were affected by the disease. Clinically ill yaks typically exhibited cough, serous to mucopurulent nasal discharge, and dyspnea; most also showed diarrhea with watery or pasty feces, some mixed with blood and mucus. Necropsy examinations of deceased yaks revealed pulmonary inflammation, enteritis with hemorrhagic lesions in the small intestine, and, in some cases, ulcerative lesions of the oral mucosa. Sampled farms had morbidity rates ranging from approximately 30% to 80% and mortality rates from 20 to 50%.

A total of 260 clinical samples were collected from 167 symptomatic yaks in 14 affected farms across Sichuan and Qinghai Provinces between January 2024 and October 2025 (sample details are shown in Table 1 and sampling locations in Supplementary Table S1). These samples included 15 lung tissues, 15 intestinal tissues, 66 serum samples, 47 nasal swabs, and 117 fecal swabs (detailed information for each sample type is provided in Supplementary Table S3). All specimens were resuspended in an appropriate volume of Dulbecco’s modified Eagle’s medium (DMEM), transported to the laboratory under refrigerated conditions, and stored at −80 °C until further analysis. All animal sampling procedures were approved by the Institutional Animal Care and Use Committee of Southwest Minzu University (approval no. 202401096), and informed consent was obtained from farm owners.

**Table 1:** Detection results of clinical samples.

Sampling site	No. of samples	No. of yaks	BVDV (%)		BoHV-1 (%)		BCoV (%)	BRVA (%)
			in samples	in yaks	in samples	in yaks		
SC-1	12	4	41.67	50	0	0	0	0
SC-2	25	7	36	71.43	0	0	0	0
SC-3	20	12	65	91.67	29	25	0	0
SC-4	20	5	50	80	45	60	0	0
SC-5	20	5	45	80	30	40	0	0
SC-6	31	15	51.61	60	0	0	0	0
SC-7	15	8	46.67	62.5	0	0	0	0
SC-8	6	6	33.33	33.33	0	0	0	0
SC-9	18	15	94.44	93.33	0	0	0	0
SC-10	21	18	80.95	88.89	0	0	0	0
QH-1	11	11	54.54	54.54	0	0	0	0
QH-2	10	10	30	30	0	0	0	0
QH-3	24	24	12.5	12.5	0	0	0	0
QH-4	27	27	33.33	33.33	0	0	0	0
Overall	260	167	48.46	55.69	8.08	4.79	0	0

### 2.2 Nucleic acid extraction and detection of pathogens

Total viral RNA was extracted from processed specimens using TRIzol™ reagent, while genomic DNA was extracted using the phenol–chloroform method, following the manufacturers’ protocols. For RNA viruses, reverse transcription was performed prior to PCR amplification. BVDV, Bovine Coronavirus (BCoV), Bovine Herpesvirus-1 (BoHV-1), and Bovine Rotavirus group A (BRVA) were detected using specific PCR/RT-PCR assays based on previously reported methods (Vilček et al., 1994, 2001; Gomez et al., 2017; Yan et al., 2020), primer information is listed in Supplementary Table S2. Preliminary genotyping of BVDV was performed based on 5′ UTR and Npro sequencing amplified from clinical samples.

### 2.3 Virus isolation and identification

BVDV-positive specimens were inoculated onto confluent monolayers of Madin-Darby bovine kidney (MDBK) cells and passaged three times, the MDBK cells and serum used in this study were confirmed to be free of BVDV contamination by RT-PCR. Isolates showing characteristic CPE were subjected to plaque purification. Viral identity was confirmed by RT-PCR, indirect immunofluorescence assay (IFA), and transmission electron microscopy (TEM). For IFA, infected cell monolayers were stained using rabbit anti-BVDV-1a (strain C24V) immune serum as the primary antibody and goat anti-rabbit IgG as the secondary antibody. TCID50 was determined by the Reed-Muench method.

### 2.4 Genome sequencing

Purified virus (1 mL) was RNase-treated, RNA extracted with TRIzol™, and resuspended in DEPC-water. Libraries were prepared and sequenced (Illumina, 2 × 150 bp, ~6 Gb depth) by Shengong Biotech (Shanghai, China). Raw reads were quality-controlled, mapped, and assembled to generate complete viral genomes.

### 2.5 Sequences and phylogenetic analysis

ORFs were identified using ORFfinder (NCBI). Multiple sequence alignments were performed with MAFFT, and similarity analyses were performed using DNAStar MegAlign. The best-fit evolutionary models were selected with ModelFinder, and maximum-likelihood (ML) phylogenetic trees were constructed in MEGA 11 with 1,000 bootstrap replicates. Pairwise distances (p-distances) were calculated following the method of Smith et al. (2017) for the full CDS (nt/aa) and NS5B (aa 3312–3837) (Smith et al., 2017). Recombination events were assessed with RDP v4.101.

To characterize molecular features of the E2 protein, homology-based three-dimensional structural models were evaluated for the study isolates (SC-H03, QH-B04) and representative reference strains of BVDV-1/2/3. The models were constructed using SWISS-MODEL with the crystal structure of the BVDV-1a NADL E2 glycoprotein (PDB ID: 4JNT) as the template (Li et al., 2013), which is currently the only experimentally resolved BVDV E2 structure available. The resulting models were visualized and structurally superimposed in PyMOL for comparative analysis.

### 2.6 Cross-neutralization assays

To evaluate antigenic relationships between the SC-H03/QH-B04 isolates and established BVDV-1/2 strains, cross-neutralization assays were performed. Positive sera against BVDV-1 (strain NM08/01) and BVDV-2 (strain AH09), prepared from whole virus and provided by Sinovet (Jiangsu) Biopharmaceuticals Co., Ltd., were tested for neutralizing activity against the study isolates as well as the reference strains BVDV-1 (NM08/01) and BVDV-2 (AH09). The antiserum against strain NM08/01 was generated using a commercial inactivated BVDV-1a vaccine.

## 3 Results

### 3.1 Pathogen detection and preliminary genotyping of BVDV

Of the 260 specimens collected from 167 affected yaks across 14 farms, 126 samples from 93 yaks tested positive for bovine viral diarrhea virus (BVDV), corresponding to an individual-level detection rate of 55.69% (93/167). Additionally, 21 samples from 8 yaks were positive for BoHV-1, while no other screened pathogens were detected. At the farm level, BVDV positivity reached 100% (14/14 farms; Table 1). Detection rates for various sample types are detailed in Supplementary Table S3, with the highest rates observed in lung tissues (73.33%) and intestinal tissues (66.67%).

**Figure 1:**
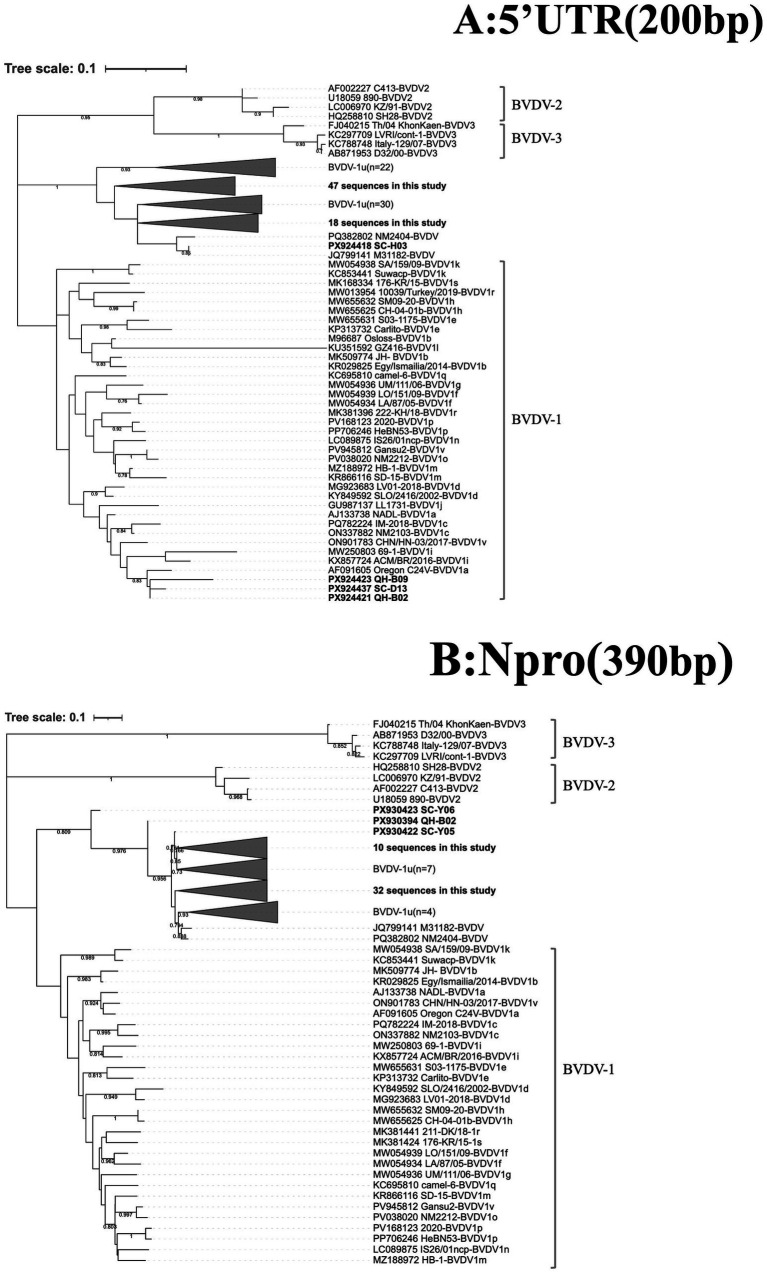
Phylogenetic analysis of BVDV isolates from yaks. **(A)** Maximum-likelihood tree inferred from the 5′ UTR region (200 bp). **(B)** Maximum-likelihood tree inferred from the Npro region (390 bp). Trees were constructed in MEGA using the best-fit nucleotide substitution model (selected in MEGA) and assessed with 1,000 bootstrap replicates. Isolates from this study are shown in bold. Bootstrap values ≥70% (1,000 replicates) are shown at the corresponding nodes. Scale bars indicate the number of nucleotide substitutions per site.

Sequencing of positive samples yielded 69 partial 5′ UTR sequences (GenBank accession nos. PX924420–PX924486) and 45 partial Npro gene sequences (GenBank accession nos. PX930393–PX930435). Representative reference sequences with well-defined genotypes were retrieved from GenBank, including all previously designated BVDV-1u sequences (52 for 5′ UTR and 11 for Npro), as well as the recently reported strain NM2404 (GenBank accession no. PQ382802; isolated from cattle in China in March 2024) and strain M31182 (GenBank accession no. JQ799141; the first BVDV genome from yaks, collected in December 2010). Following multiple sequence alignment, phylogenetic trees were constructed using the ML method with the best-fit nucleotide substitution model (as determined by ModelTest). For the 5′ UTR (200 bp), 66/69 sequences from this study formed a distinct monophyletic clade with strong bootstrap support. This clade clustered closely with NM2404, M31182, and partial 5′ UTR sequences previously assigned to BVDV-1u. These 66 sequences originated from 14 farms; the remaining 3/69 5′ UTR sequences clustered within the established BVDV-1a subgenotype (Figure 1A). Meanwhile, Phylogenetic analysis of the Npro fragment (390 bp) placed all 45 sequences from 10 farms in the same independent monophyletic clade alongside M31182, NM2404, and the BVDV-1u reference sequences (Figure 1B).

### 3.2 Virus isolation and identification

**Figure 2:**
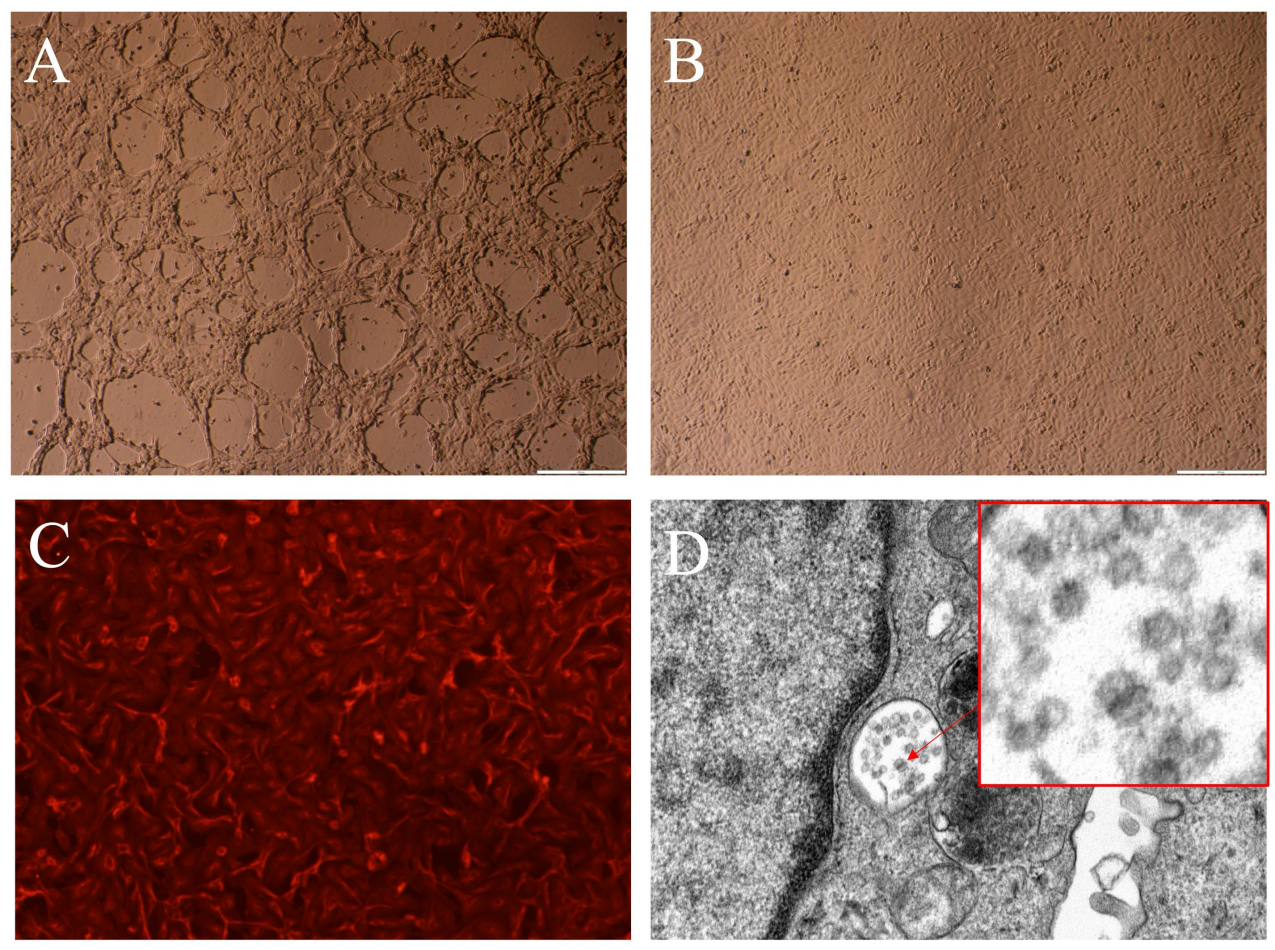
CPE and identification of BVDV isolates in MDBK cells. **(A)** Typical CPE observed in MDBK cells at 90 hpi, characterized by cell rounding, cytoplasmic granulation, cell detachment, and enlargement of intercellular spaces. **(B)** Uninfected control cells after 90 h of culture. **(C)** IFA of infected cells using a rabbit polyclonal anti-BVDV-1a (C24V) immune serum; widespread fluorescence indicates viral antigen expression. **(D)** TEM image showing pestivirus-like particles (~40–60 nm) in the cytoplasm; inset shows a higher-magnification view.

Samples clustering in an independent phylogenetic branch based on 5’ UTR and Npro sequences were inoculated onto MDBK cell monolayers for virus isolation. First-passage cultures showing CPE were subjected to three additional passages and plaque purification, yielding four CP BVDV isolates: QH-B04, QH-B10, SC-H03, and SC-L17, their TCID50 values were determined as 10^6.0^, 10^7.0^, 10^7.0^, and 10^7.5^ TCID50/0.1 mL, respectively, using the Reed–Muench method. CPE was observed at 80–90 hours post-inoculation (hpi) and intensified by 120–144 hpi, characterized by cell rounding, cytoplasmic granulation, and widened intercellular spaces (Figure 2A). In contrast, uninfected MDBK cells maintained normal morphology and served as the negative control (Figure 2B). RT-PCR confirmed the isolates as BVDV; 5′ UTR and Npro sequences from the isolates matched the independent phylogenetic branch observed in sequence-based analyses. IFA and TEM further confirmed viral antigen distribution and virion morphology, respectively (Figures 2C,D).

### 3.3 Genomic characteristics of the isolates

Metagenomic sequencing generated two genome sequences with lengths of 12,186 bp (SC-H03) and 12,407 bp (QH-B04), each containing a full ORF of 11,709 bp and 11,706 bp, respectively. The genome organization is illustrated in Supplementary Figure S1. These sequences have been deposited in GenBank under accession numbers PX924418 (SC-H03) and PX924419 (QH-B04). The nucleotide identity between the two isolates was 91.5%. BLAST comparisons against all 325 BVDV genomes in GenBank (retrieved January 1, 2026) indicated the highest similarities to strains NM2404 and M31182, ranging from 82.3% to 93.6%. At the complete coding sequence (CDS) level, the highest nucleotide similarities between our isolates and well-defined BVDV-1/2/3 genomes were approximately 77.8%, 75.1%, and 73.9%, respectively. In contrast, similarities to other recognized Pestivirus species were markedly lower, ranging from 47.9% to 73.6% (Supplementary Table S4).

**Figure 3:**
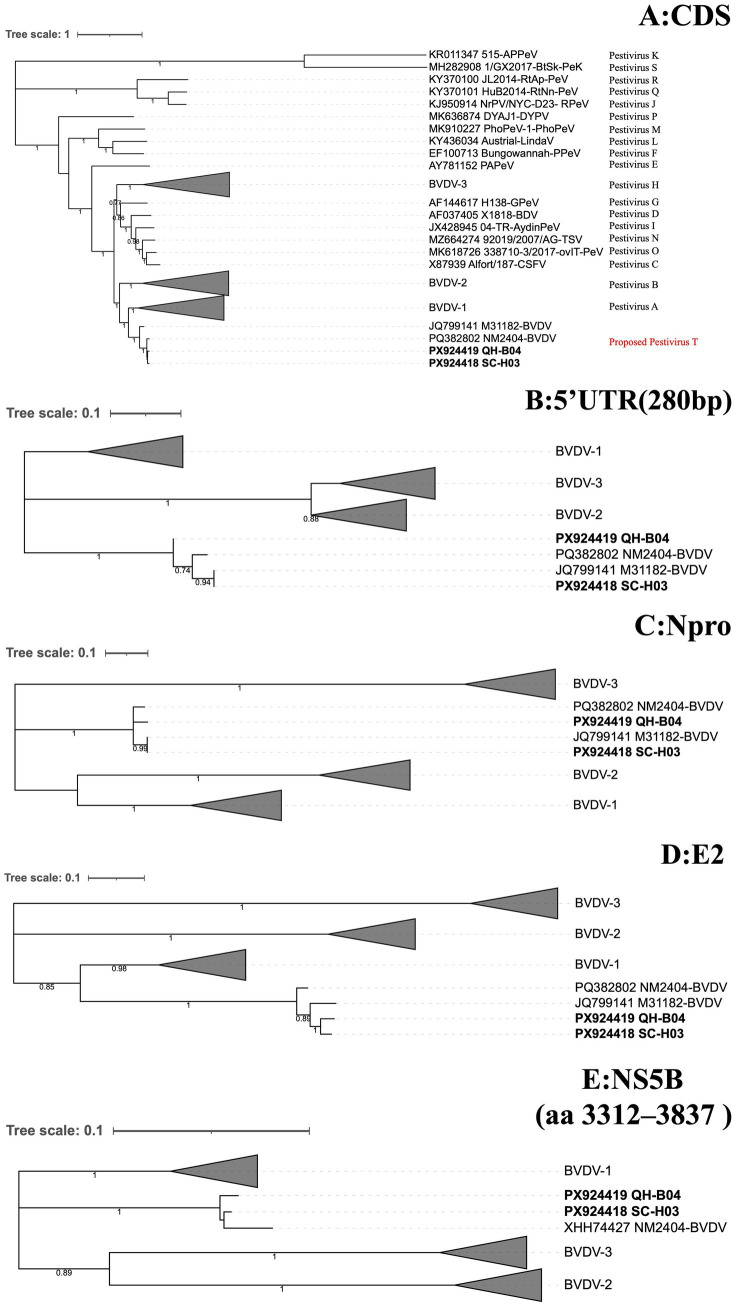
Phylogenetic relationships of the isolates with representative pestiviruses based on multiple genomic regions. **(A)** ML tree based on the CDS nucleotide sequences (GTR + G + I). **(B)** ML tree based on the 5′ UTR (280 bp) nucleotide sequences (K2 + G + I). **(C)** ML tree based on the Npro nucleotide sequences (GTR + G + I). **(D)** ML tree based on the E2 nucleotide sequences (GTR + G + I). **(E)** ML tree based on the conserved NS5B (RdRp) amino acid sequences (aa 3312–3837; LT + G + I). Bootstrap support values were estimated using 1,000 replicates. The numbers of reference sequences included for BVDV-1, BVDV-2, and BVDV-3 were 226, 69, and 28, respectively. Isolates obtained in this study are shown in bold. Only bootstrap values ≥70% are displayed at the corresponding nodes. Scale bars indicate the number of substitutions per site.

After selecting the best-fit model, ML phylogenetic analysis based on complete CDS nucleotide sequences showed that QH-B04 and SC-H03 clustered together with M31182 and NM2404 to form a distinct clade supported by a bootstrap value of 100%. The phylogenetic tree included 226, 69, and 28 representative reference sequences of BVDV-1, BVDV-2, and BVDV-3, respectively. This clade was clearly separated from the established BVDV-1/2/3 clades (Figure 3A). Phylogenetic trees based on individual genes exhibited consistent topologies, with SC-H03 and QH-B04 grouping in a single monophyletic branch alongside M31182 and NM2404, supported by bootstrap values >80% and distinct from recognized BVDV lineages (Figures 3B–E; Supplementary Figure S2).

No recombination events were detected by RDP4 (v4.101) using multiple detection methods.

P-distance analyses were conducted for the complete CDS (nt and aa) and NS5B (RdRp, aa 3312–3837) regions, according to ICTV-recommended criteria for pestivirus classification (Smith et al., 2017). The p-distances between the isolates identified in this study and representative strains of the 19 recognized Pestivirus species are summarized in Table 2. In addition, the maximum intra-species genetic distances observed within BVDV-1, BVDV-2, and BVDV-3 are shown in Table 3.

**Table 2:** Minimum pairwise p-distances between isolates from this study and representative strains of the 19 Pestivirus species across key genomic regions.

Species	CDS (nt)	CDS (aa)	NS5B (aa 3312–3837)
Pestivirus A	0.2740	0.2004	0.1503
Pestivirus B	0.3032	0.2423	0.1986
Pestivirus C	0.3325	0.2818	0.2366
Pestivirus D	0.3314	0.2809	0.2280
Pestivirus E	0.3951	0.4071	0.2953
Pestivirus F	0.4314	0.4627	0.3426
Pestivirus G	0.3247	0.2775	0.2228
Pestivirus H	0.3219	0.2705	0.1986
Pestivirus I	0.3245	0.2756	0.2176
Pestivirus J	0.4996	0.5882	0.4523
Pestivirus K	0.5218	0.6403	0.4764
Pestivirus L	0.4353	0.4681	0.3476
Pestivirus M	0.4312	0.4723	0.3443
Pestivirus N	0.3312	0.2784	0.2159
Pestivirus O	0.3245	0.2795	0.2278
Pestivirus P	0.4382	0.4821	0.3737
Pestivirus Q	0.4966	0.5903	0.4506
Pestivirus R	0.5036	0.6005	0.4531
Pestivirus S	0.5238	0.6409	0.4781

**Table 3:** Maximum *p*-distances among members of individual BVDV genotypes across key genomic regions.

Species	CDS (nt)	CDS (aa)	NS5B (aa 3312–3837)
Pestivirus A (*n* = 28)	0.2200	0.1426	0.1174
Pestivirus B (*n* = 4)	0.0901	0.0678	0.0587
Pestivirus H (*n* = 4)	0.0949	0.0673	0.0415

### 3.4 E2 protein analysis

The E2 gene is 1,122 bp in length and encodes a 374 amino acid glycoprotein. At the amino-acid level, SC-H03 and QH-B04 differed from the BVDV-1a reference strain NADL (GenBank accession no. AJ133738) by 105 residues (28.1%), from the BVDV-1b strain Osloss (GenBank accession no. M96687) by 109 residues (29.1%), from the BVDV-2 reference strain 890 (GenBank accession no. U18059) by 136 residues (36.4%), and from the BVDV-3 strain D32/00 (GenBank accession no. AB871953) by 145 residues (38.8%) (Supplementary Figure S3).

**Figure 4:**
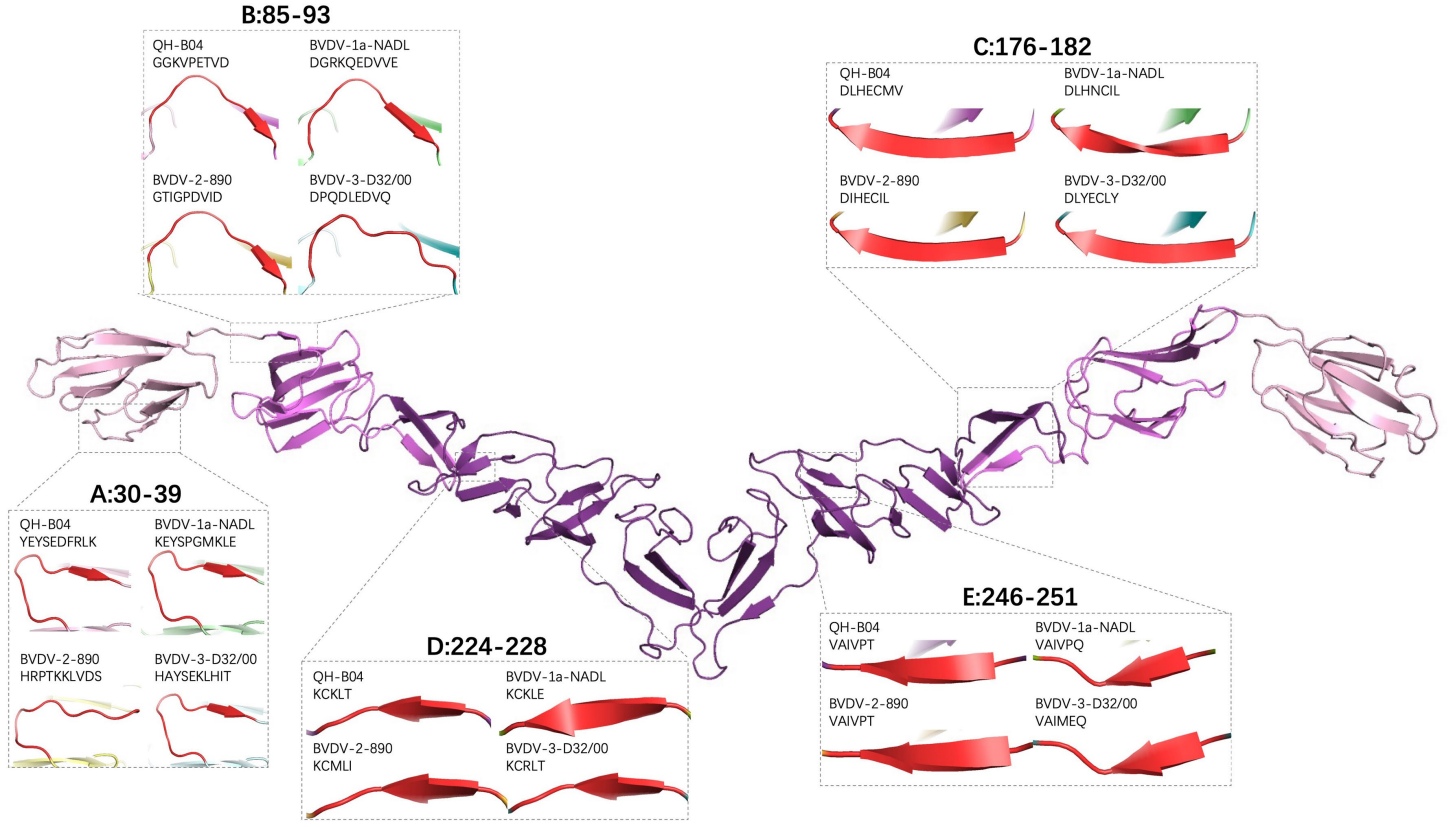
Homology modeling of the E2 protein three-dimensional structure using SWISS-MODEL with PDB ID: 4JNT as the template. Five major divergent regions are highlighted: **A** (aa 30–39), **B** (aa 85–93), **C** (aa 176–182), **D** (aa 224–228), and **E** (aa 246–251).

Homology modeling of E2 using SWISS-Model with PDB template 4JNT (BVDV-1 E2 crystal structure) and subsequent structural comparison (QH-B04 shown as representative) revealed pronounced conformational differences in five functionally important regions: aa 30–39, 85–93, 176–182, 224–228, and 246–251 (Figures 4A–E). Since the homology modeling was based on the sole currently available BVDV E2 structure modeling derived from a BVDV-1a strain, the structural differences derived from the comparison require further experimental validation.

### 3.5 Cross-neutralization assays

To better understand the antigenic relationships between the strain SC-H03 in this study and BVDV-1/2 genotypes, cross-neutralization assays were performed using positive sera against BVDV-1 (strain NM08/01) and BVDV-2 (strain AH09), and the results are summarized in Table 4.

**Table 4:** Cross-neutralization assay between reference BVDV-1/2 strains and the isolate in this study.

Antiserum	Virus	1:32	1:64	1:128	1:256	1:512	1:1024	1:2048
BVDV-1	NM08/01	−	−	−	−	−	−	−
	SC-H03	+	+	+	+	+	−	−
BVDV-2	AH09	−	−	−	−	−	−	−
	SC-H03	+	+	+	+	+	+	+

“−” indicates detectable virus neutralization, whereas “+” indicates absence of neutralizing activity.

1:X indicates the serum neutralizing titer, with each titer tested in quadruplicate.

## 4 Discussion

The taxonomy of the genus Pestivirus is primarily based on phylogenetic analyses and pairwise nucleotide sequence distance calculations of the CDS, complemented by antigenic properties, natural host range, and pathological features(Smith et al., 2017; Postel et al., 2021). Currently, the genus Pestivirus comprises 19 species, including three BVDV species: Pestivirus A (BVDV-1), Pestivirus B (BVDV-2), and Pestivirus H (BVDV-3) (Genus: Pestivirus | ICTV). In this study, we obtained two genomes from isolates SC-H03 and QH-B04, which shared the highest nucleotide and amino acid similarities with strains M31182 (GenBank accession No. JQ799141) and NM2404 (GenBank accession No. PQ382802), but exhibited significant divergence from other BVDV-1/2/3 sequences. Moreover, phylogenetic analyses based on the complete CDS and each individual gene consistently placed SC-H03 and QH-B04 together with M31182 and NM2404 in a strongly supported monophyletic clade, distinct from the established BVDV-1/2/3 clades. P-distance analyses further corroborated this topology: the minimum p-distances between our isolates and the 19 recognized Pestivirus species exceeded the maximum intra-species p-distances observed within BVDV-1/2/3 in this study (Tables 2, 3); and also surpassed the minimum inter-species p-distances reported for Pestivirus members isolated from the same host species by Postel et al. (2021). Notably, the minimum p-distances for the CDS (nt/aa) between Pestivirus O and Pestivirus N are 0.23/0.14, and for NS5B (3312–3837) is 0.11, whereas the minimum p-distances between the strains in this study and the 19 Pestivirus genus members are 0.27/0.20 for the CDS (nt/aa) and 0.15 for NS5B (3312–3837). Furthermore, cross-neutralization assays demonstrated significant antigenic differences between the strains in this study and representative BVDV-1 and BVDV-2 strains. Although this study lacked cross-neutralization test with BVDV-3 strain, the substantial differences in genomic characteristics are sufficient to determine that they do not belong to the same genotype. Taken together, these findings are consistent with the ICTV species demarcation criteria for the genus Pestivirus and support the proposal that SC-H03 and QH-B04 represent a potential new BVDV genotype, which we propose to designate as BVDV-4 (Pestivirus T).

Interestingly, two GenBank-recorded strains, NM2404 and M31182, showed a close genetic relationship with our isolates. NM2404 was annotated as BVDV-1v which was first proposed in 2020 (Deng et al., 2020). To date, five BVDV-1v genome sequences have been reported, including strain NM2404 and four additional isolates (GenBank accession nos. PV945812, ON901783, ON901784, and ON901785) (Zhu et al., 2022). However, NM2404 exhibited only 75.0%–75.4% nucleotide identity to the four reported BVDV-1v genomes, while sharing 93.8% nucleotide identity with SC-H03 and QH-B04 across the complete CDS. Furthermore, phylogenetic analyses consistently placed NM2404 together with SC-H03 and QH-B04 in a distinct, well-supported monophyletic lineage, separate from the established BVDV-1v cluster (Figure 1). Similarly, strain M31182, deposited in GenBank in 2012 and representing the first yak-derived BVDV genome, was previously classified as an unclassified genotype (Sun, 2012), and no subsequent studies have further characterized this lineage. Taken together, our results suggest that NM2404 and M31182 should also be classified within the same potential new BVDV genotype as SC-H03 and QH-B04, which we propose to designate as BVDV-4 (Pestivirus T).

Phylogenetic analyses based on 5′ UTR and Npro fragments grouped SC-H03 and QH-B04 with previously reported “BVDV-1u” sequences (Deng et al., 2015; Li et al., 2025a). The subtype BVDV-1u was first proposed based on partial 5′ UTR sequences approximately 200 bp (Deng et al., 2015), and this designation has been cited in several subsequent studies (Yeşilbağ et al., 2017; Zhang et al., 2022; Li et al., 2025a; Rana et al., 2025a). However, to date, no complete genome sequences, key typing genes [such as full-length Npro, E2, and NS5B (RdRp; aa 3312–3837)], or successful virus isolates have been reported for BVDV-1u strains. As noted previously, phylogenies inferred from short genomic regions such as the 5′ UTR or Npro often suffer from limited resolution and inconsistent branching patterns, whereas analyses of complete Npro, NS5B (RdRp; aa 3312–3837), and E2 coding sequences can provide high confidence in assigning BVDV isolates to established or novel subgenotypes (Yeşilbağ et al., 2017; Al-Kubati et al., 2021). In contrast, our analyses based on the complete CDS and multiple coding regions provide robust and consistent evidence that SC-H03, QH-B04, NM2404, and M31182 constitute a distinct evolutionary lineage, separated from BVDV-1/2/3 at inter-species genetic distances. These results suggest that previously reported “BVDV-1u” sequences may represent partial fragments of the potential new BVDV genotype identified in this study. However, additional genomes are needed to confirm this speculation.

The E2 protein is a key determinant mediating receptor binding, viral entry, cellular tropism, and host range, and is also the main antigen inducing neutralizing antibody production, playing a central role in BVDV pathogenesis and immunity (Wang et al., 2015). Our analysis showed significant amino-acid heterogeneity between the E2 proteins of SC-H03/QH-B04 and BVDV-1/2/3 (shown in Supplementary Figure S3), resulting in predicted conformational changes. Notably, most substitutions were concentrated in the N-terminal region of E2, which harbors the receptor-binding domain and contains the *β*-hairpin structure implicated in virus–receptor interactions (Merwaiss et al., 2021; Qi et al., 2022), this region is also enriched in neutralizing epitopes (Li et al., 2013). In this study, the β-hairpin structure of SC-H03/QH-B04 was predicted to be located at residues 136–158, which contains four genotype-specific amino acid substitutions, while previous studies have shown that amino acid mutations in this region may modulate the E2–receptor interaction interface, thereby influencing receptor usage, viral entry efficiency, and cellular tropism (Liang et al., 2003; El Omari et al., 2013). Furthermore, the well-characterized neutralizing epitope located at residues 71–77 of E2 is known to be immunologically critical, in which residue 72 directly contributes to neutralizing antibody binding (Paton et al., 1992; Deregt et al., 1998). Interestingly, residue 72 is a positively charged arginine in BVDV-1/2/3, while this residue was replaced by a negatively charged aspartic acid in SC-H03/QH-B04, which may facilitate antigenic drift and immune escape (Paton et al., 1992).

In this study, 55.69% BVDV positivity was detected in 167 diseased yaks from 14 farms, and the dominant epidemic strains (95.7%) were the potential new genotype of BVDV based on 5′ UTR and Npro sequencing amplified from clinical samples; farm-level positivity was 100%, and the two most geographically distant affected farms were separated by more than 900 km. Thus, we can conclude that the novel BVDV has been widely spreading across Sichuan and Qinghai provinces. It is worth noting that high morbidity and mortality rates occurred in affected farms, causing serious economic losses, which were similar to those during the early emergence of BVDV-2 and BVDV-3 (Pellerin et al., 1994; Decaro, 2020). Moreover, clinical manifestations and high viral detection rates in lung (73.3%) and intestinal tissues (66.7%) in affected yaks (Supplementary Table S1) indicated that the novel BVDV was associated with the disease outbreak. In fact, in addition to the direct harm caused by BVDV, it often predisposes yaks to secondary infections from opportunistic bacterial or viral pathogens such as Pasteurella due to mucosal damage and immune suppression, thereby increasing the severity and mortality rate of the disease (Potgieter, 1995; Ridpath, 2010). Further artificial infection experiments are necessary to better understand the pathogenic characteristics of the novel BVDV. It is concerning that this novel BVDV remains prevalent, whereas the currently licensed BVDV vaccines used in China are based on BVDV-1 strains. Based on the cross-neutralization results, these vaccines are likely to provide limited cross-protection against the novel BVDV.

BVDV is capable of infecting a broad range of domestic and wild hosts, and persistently infected animals serve as long-term reservoirs that sustain viral circulation and facilitate transmission across populations (Potgieter, 1995; Ridpath, 2010). As a unique bovine species native to the Qinghai–Tibetan Plateau, yaks are predominantly raised under free-grazing systems with extensive movement ranges and frequently share habitats with sheep and diverse wildlife species, including deer, wild goats, other small ruminants, wild boars, and rodents. Such multi-host ecological settings provide ample opportunities for interspecies transmission, viral maintenance, and long-term evolutionary diversification (Yeşilbağ et al., 2017; Postel et al., 2021; Rana et al., 2025b). Therefore, expanded surveillance across co-grazing domestic animals and sympatric wildlife species is essential to elucidate the ecological dynamics, host range, and spillover risks associated with this emerging BVDV lineage.

## 5 Conclusion

In summary, we identified a potential new BVDV genotype that is emerging and prevalent in yaks; its genome sequences and antigenicity are significantly distinct from existing BVDV-1/2/3. In accordance with ICTV classification criteria for the genus Pestivirus, it meets the criteria to be classified as a new species in the genus Pestivirus, which we propose to designate as BVDV-4 (Pestivirus T). This virus has been widely circulating in yaks across Sichuan and Qinghai provinces in China, and is associated with severe outbreaks. Our findings expand the understanding of BVDV genetic diversity and highlight the urgent need for development of effective control strategies targeting this potential new BVDV genotype.

## Data Availability

The datasets presented in this study can be found in online repositories. The names of the repository/repositories and accession number(s) can be found in the article/Supplementary material.

## Glossary

BVDV: Bovine Viral Diarrhea Virus
5′ UTR: 5′ untranslated region
CDS: Complete coding sequence
ICTV: International Committee on Taxonomy of Viruses
CSFV: Classical Swine Fever Virus
WOAH: World Organisation for Animal Health
PI: Persistently infected
CPE: Cytopathic effects
Cp: Cytopathic
ncp: Non-cytopathic
ORF: Open reading frame
DMEM: Dulbecco’s modified Eagle’s medium
BCoV: Bovine Coronavirus
BoHV-1: Bovine Herpesvirus-1
BRVA: Bovine Rotavirus group A
MDBK: Madin-Darby bovine kidney
IFA: Indirect immunofluorescence assay
TEM: Transmission electron microscopy
ML: Maximum-Likelihood
p-distances: Pairwise distances
hpi: hours post-inoculation
