# Supplementary material for: Isolation and identification of a potential new bovine viral diarrhea virus genotype associated with severe outbreaks in yaks (Bos grunniens) in China
Source: Front Microbiol. 2026 Jul 22;17:1908819. doi: 10.3389/fmicb.2026.1908819 (PMC13438301; doi:10.3389/fmicb.2026.1908819)
Supplement: Supplementary file 1 [file Table_1.DOCX]

Supplementary Table S1. Geographic sampling details of yak farms included in this study.

| Province | County | Longitude | Latitude |
| --- | --- | --- | --- |
| SiChuan | Seda | 100.05 | 32.25 |
| SiChuan | Yajiang | 101.35 | 30 |
| SiChuan | Litang | 100.25 | 30 |
| SiChuan | Shiqu | 98.4 | 33.1 |
| SiChuan | Danba | 101.85 | 30.85 |
| SiChuan | Hongyuan | 102.6 | 32.75 |
| QingHai | Qilian | 100.2 | 38 |
| QingHai | Henan | 101.95 | 34.65 |
| QingHai | Zaduo | 94.85 | 32.7 |
| QingHai | Zhiduo | 95.8 | 34.6 |

Supplementary Table S2. Primer information for pathogen detection.

| Pathogens | Primer Name | Sequence(5'-3') | Size (bp) | Target Gene | Reference |
| --- | --- | --- | --- | --- | --- |
| BVDV | 324-F | ATGCCCWATTAGTAGGACTAGC | 288 | 5'UTR | Vilček S, et al.,1994. |
|  | 326-R | TCAACTCCATGTGCCATGTAC |  |  |  |
|  | Npro-F | TCTCTGCTGTACATGGCACATG | 428 | Npro | Vilček S, et al.,2001. |
|  | Npro-R | CCATCTATRCACACATAAATGTGGT |  |  |  |
| BCoV | BCoV-F | CTAGTAACCAGGCTGATGTCAATACC | 81 | N | Gome, D.E. et al., 2017 |
|  | BCoV-R | GGCGGAAACCTAGTCGGAATA |  |  |  |
| BoHV-1 | BHV-F | ATGCCGCGATACAACTACACTGAAC | 921 | gD | WOAH Terrestrial Manual |
|  | BHV-R | TTATTCGAGGCTCGGCCAGCCTT |  |  |  |
| BRVA | BRVA-F | CCACCAGGTATGAATTGGAC | 231 | VP6 | Yan N et al., 2020 |
|  | BRVA-R | GAGTAATCACTCAGATGGCG |  |  |  |

Supplementary Table S3. Detection results of BVDV in different types of clinical samples

| Sampling site | Nasal swab | Intestinal swab | Blood | Lung | Intestinal |
| --- | --- | --- | --- | --- | --- |
| SC-1 | 4 | 5 | 3 |  |  |
| SC-2 | 4 | 8 | 3 | 5 | 5 |
| SC-3 | 1 | 11 | 8 |  |  |
| SC-4 | 5 | 10 | 5 |  |  |
| SC-5 | 5 | 10 | 5 |  |  |
| SC-6 | 8 | 3 | 6 | 7 | 7 |
| SC-7 | 3 | 2 | 6 | 2 | 2 |
| SC-8 |  |  | 6 |  |  |
| SC-9 | 2 | 2 | 12 | 1 | 1 |
| SC-10 | 6 | 3 | 12 |  |  |
| QH-1 |  | 11 |  |  |  |
| QH-2 | 9 | 1 |  |  |  |
| QH-3 |  | 24 |  |  |  |
| QH-4 |  | 27 |  |  |  |
| Overall | 47 | 117 | 66 | 15 | 15 |
| BVDV Positively | 57.45% | 33.33% | 59.09% | 73.33% | 66.67% |

Supplementary Figure S1. Genome organization of isolates characterized in this study.

Supplementary Table S4. Pairwise nucleotide identity of complete CDS among pestiviruses

| Pestivirus Group | Intraspecies (%) | With Isolates from This Study (%) |
| --- | --- | --- |
| Pestivirus A | 80.2-97.7 | 76.4-77.8 |
| Pestivirus B | 88.8-95.5 | 72.5-75.1 |
| Pestivirus C | - | 72.9-73.0 |
| Pestivirus D | - | 73.0-73.1 |
| Pestivirus E | - | 66.9-67.0 |
| Pestivirus F | - | 63.5 |
| Pestivirus G | - | 73.0-73.1 |
| Pestivirus H | 92.3-96.6 | 73.5-73.9 |
| Pestivirus I | - | 73.5-73.6 |
| Pestivirus J | - | 53.7 |
| Pestivirus K | - | 48.2-48.3 |
| Pestivirus L | - | 63.5-63.6 |
| Pestivirus M | - | 62.4 |
| Pestivirus N | - | 73.1 |
| Pestivirus O | - | 73.6 |
| Pestivirus P | - | 62.6 |
| Pestivirus Q | - | 53.8-53.9 |
| Pestivirus R | - | 53.5-53.7 |
| Pestivirus S | - | 47.9-48.1 |
| Proposed Pestivirus T | 94.6-96 | - |

Supplementary Figure S2. Phylogenetic analyses based on 7 genes of BVDV

Note: Trees were constructed in MEGA using the best-fit nucleotide-substitution model (selected in MEGA) and assessed with 1,000 bootstrap replicates. Isolates from this study are shown in bold. Bootstrap values ≥70% (1,000 replicates) are shown at the corresponding nodes. Scale bars indicate the number of nucleotide substitutions per site.

maximum-likelihood phylogenetic analysis of 7 genes showed that QH-B04 and SC-H03, along with M31182 and NM2404, clustered to form a distinct clade with strong bootstrap support (>80%).

Supplementary Figure S3. Multiple sequence alignment of E2 amino acid sequence
